# T Cell Bispecific Antibodies: An Antibody-Based Delivery System for Inducing Antitumor Immunity

**DOI:** 10.3390/ph14111172

**Published:** 2021-11-17

**Authors:** Daisuke Kamakura, Ryutaro Asano, Masahiro Yasunaga

**Affiliations:** 1 Division of Developmental Therapeutics, Exploratory Oncology Research & Clinical Trial Center, National Cancer Center, Kashiwa 277-8577, Japan; d.kamakura1019@gmail.com; 2 Department on Biotechnology and Life Science, Graduate School of Engineering, Tokyo University of Agriculture and Technology, Tokyo 184-8588, Japan; ryutaroa@cc.tuat.ac.jp

**Keywords:** T cell bispecific antibody, T-BsAb, pharmacokinetics, T cell redirection, mechanism of action, drug development

## Abstract

As a breakthrough immunotherapy, T cell bispecific antibodies (T-BsAbs) are a promising antibody therapy for various kinds of cancer. In general, T-BsAbs have dual-binding specificity to a tumor-associated antigen and a CD3 subunit forming a complex with the TCR. This enables T-BsAbs to crosslink tumor cells and T cells, inducing T cell activation and subsequent tumor cell death. Unlike immune checkpoint inhibitors, which release the brake of the immune system, T-BsAbs serve as an accelerator of T cells by stimulating their immune response via CD3 engagement. Therefore, they can actively redirect host immunity toward tumors, including T cell recruitment from the periphery to the tumor site and immunological synapse formation between tumor cells and T cells. Although the low immunogenicity of solid tumors increases the challenge of cancer immunotherapy, T-BsAbs capable of immune redirection can greatly benefit patients with such tumors. To investigate the detailed relationship between T-BsAbs delivery and their T cell redirection activity, it is necessary to determine how T-BsAbs deliver antitumor immunity to the tumor site and bring about tumor cell death. This review article discusses T-BsAb properties, specifically their pharmacokinetics, redirection of anticancer immunity, and local mechanism of action within tumor tissues, and discuss further challenges to expediting T-BsAb development.

## 1. Introduction

In the last two decades, cancer immunotherapy has been developed as the fourth pillar of cancer therapy, in addition to surgery, radiation, and chemotherapy [1]. Cancer immunotherapies are designed to exploit host immunity and eliminate tumors either by promoting the antitumor immune system or by suppressing immune inhibitory factors. Many types of immune cells, such as T cells, NK cells, and dendritic cells, are associated with the immune response, and their effector functions are utilized to bring about tumor eradication. Among them, T cells are the central component of adaptive immunity and have been most commonly applied due to their potent cytotoxicity and abundance in blood. Indeed, increasing therapeutic agents that redirect T cell cytotoxicity to tumor cells have achieved great success in clinical practice [2,3,4,5,6].

The most successful immunotherapy modality is antibody therapeutics, which is characterized by antibodies that block immune inhibitory receptors (e.g., programmed cell death 1 (PD-1) and cytotoxic T lymphocyte antigen 4 (CTLA-4)) or ligands (e.g., PD-L1). These so-called immune checkpoint inhibitory antibodies (CPIs) have been approved for the treatment of various cancers, including unresectable or metastatic melanoma, metastatic non-small cell lung cancer, and colorectal cancer with microsatellite instability [7,8,9]. Moreover, combination therapies with various CPIs have yielded positive outcomes thus far [10,11,12]. Although these agents have shown remarkable effectiveness for particular indications, the number of patients who benefit from these treatments is very limited. This is because the efficacy of CPIs is likely dependent on the degree of T cell infiltration within tumor tissues during the pre-treatment stage. Supporting this idea, the relevance of immune cell infiltration to the response to CPIs was demonstrated [13,14]. In addition, less effectiveness of CPI therapy against T cell-excluded tumors was reported in various types of cancer, highlighting the importance of T cell infiltration [15,16,17]. To overcome this problem, a novel immunotherapy that actively promotes T cell infiltration into tumors is required.

Genetically engineered T cell therapies that are specific for tumor cells are an emerging approach to eliminate tumors with low T cell infiltration. T cell receptor-engineered T cells (TCR-T) and chimeric antigen receptor T cells (CAR-T) are designed to selectively engage a specific neoantigen presented on major histocompatibility complex (MHC) molecules or a specific tumor-associated antigen (TAA), respectively, on tumor cells [18]. These tumor-specific T cells actively migrate to the tumor mass and kill the engaged tumor cells [19,20]. Aside from adoptive T cell transfer therapy, another technology that evokes T cell infiltration is T cell bispecific antibody (T-BsAb) therapy [21]. T-BsAbs are typically composed of two antigen-binding sites capable of recognizing either a TAA on tumor cells or a CD3 subunit forming a complex with the TCR on T cells. This simultaneous binding to two antigens induces crosslinking between tumor cells and T cells, allowing T cells to recognize the tumor cells independently of MHC engagement [22]. In contrast with CPIs, which block inhibitory signals against effector T cells, T-BsAbs can directly and preferentially activate memory T cells, and presumably to a lesser extent, naïve T cells [23,24]. Therefore, it is thought that T-BsAbs promote the redirection of host immunity toward solid tumors with low immunogenicity, a process that includes T cell recruitment and immunological synapse (IS) formation.

Despite the promise of this approach, no regulatory authorities worldwide have approved T-BsAbs for the treatment of solid cancers [25]. One reason is that T-BsAbs show insufficient clinical efficacy due to the complexity of the immune response to solid tumors. In fact, the distinctive pharmacokinetics (PK) of T-BsAbs, which result from their multispecificity, may make it difficult to understand the mechanism of T-BsAb-induced T cell regulation.

**Figure 1:**
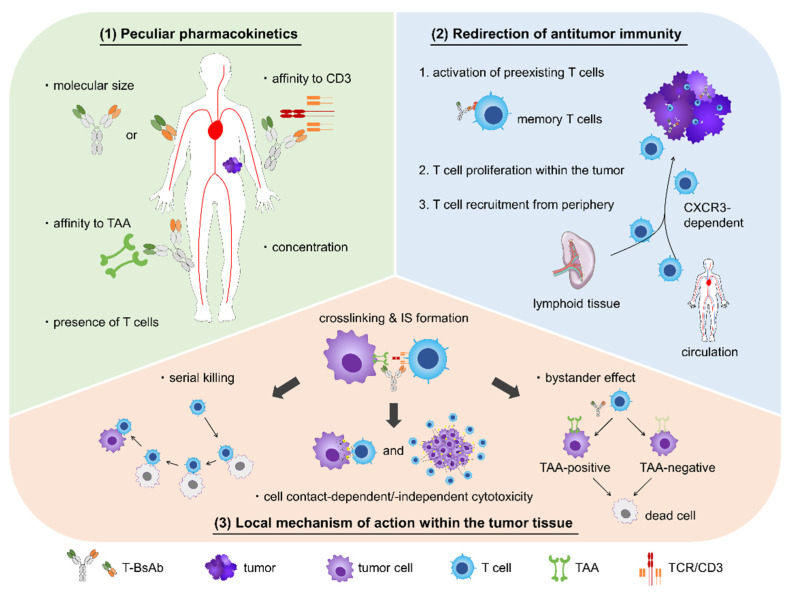
Three pharmacological properties of T-BsAbs. (1) T-BsAbs are characterized by their unique pharmacokinetics, derived from the dual binding affinity to molecules on tumor cells (tumor-associated antigens (TAA)) and T cells (CD3). As with conventional antibody therapeutics, T-BsAbs have absorption, distribution, metabolism, and excretion characteristics that are dependent on their molecular size and concentration. Furthermore, their delivery to the tumor lesion is affected by their binding affinity to not only TAA but also CD3. Within the tumor tissue, T-BsAbs are distributed partly according to the presence and location of infiltrating T cells. (2) T-BsAbs promote antitumor immunity within the tumor tissue. First, memory T cells, which are mixture of central memory and effector memory phenotypes and preexist in the tumor, are cross-linked to tumor cells by tumor-accumulated T-BsAbs, thus initiating T cell activation. Activated T cells then proliferate in response to intracellular signaling and secrete proinflammatory cytokines. Subsequently, peripheral T cells are recruited to the tumor from the circulation and secondary lymphoid tissues in a CXCR3-dependent manner. (3) Once T cells are crosslinked with tumor cells via T-BsAbs, immune synapses (ISs) are formed between pairs of cells, and activated T cells lyse the bridged tumor cells using perforin and granzyme. Active T cells continue to kill neighboring tumor cells by repeated cell contact. Moreover, T-BsAb-activated T cells release cytotoxic and proinflammatory cytokines, resulting in cell contact-independent cell killing and the FasL-mediated bystander effect, respectively.

Thus, it is necessary to explore the complex relationship between an individual BsAb agent, immunity, and cancers. From this viewpoint, we highlight the following three properties of T-BsAbs: (1) their unique PK, (2) their redirection of antitumor immunity, and (3) their local mechanism of action within tumor tissues (Figure 1). Here, we summarize previous studies related to the aforementioned elements, discuss the current status of T-BsAbs development in both preclinical and clinical stages, and point out further challenges for the development of innovative immune delivery systems.

## 2. Pharmacology of T-BsAbs

### 2.1. Unique PK

Antibody therapeutics generally have a high molecular weight (>150 kDa). Due to their large size, they tend to leak into tumor tissues from blood vessels and are then retained there since the tumor microenvironment is composed of incomplete and vulnerable vessels. This phenomenon is referred to as the enhanced permeability and retention (EPR) effect, and it plays an important role in the passive targeting of tumors [26]. Antibody therapeutics may also involve active targeting, in which agents are actively accumulated in tumor tissues due to potent affinity for TAAs. Although T-BsAbs are a form of antibody therapy, their pharmacokinetic profiles differ from those of conventional antibody agents because they have two kinds of binding domains, namely, an anti-TAA domain and an anti-CD3 domain. This makes it more difficult to determine how to achieve efficient tumor accumulation of T-BsAbs. The parameters that affect the PK of T-BsAbs include their molecular size, affinity to TAA, affinity to CD3, concentration, and presence of T cells in the tumor microenvironment.

Molecular size is the most influential factor in the PK of antibody agents [27,28,29,30]. In T-BsAbs, the complex of the TAA-binding domain(s) and the CD3-binding domain(s) is a recombinant protein, and thus a target molecular size and weight can be aimed for when generating original T-BsAbs. Generally, the size of the T-BsAb format is categorized by whether or not the agent contains an Fc fragment [31]. The most clinically successful T-BsAb format, BiTE, is composed of two Fv domains binding a TAA and a CD3, without an Fc domain [32]. Other T-BsAbs characterized by the lack of an Fc fragment include DART and diabody [33,34,35]. These Fc-lacking formats were originally produced to avoid off-target T cell activation via crosslinking between a T cell and an FcγR-positive cell. Due to their small size, however, BiTEs must be administrated very frequently [36,37,38,39]. In addition, the short half-life of T-BsAbs seems to be a drawback, especially against solid tumors. Whereas T-BsAbs are able to crosslink a tumor cell and a T cell during circulation in hematological cancers, T-BsAbs have to be delivered to solid tumor tissues from the circulation, and then bridge the two types of cells within the tumor. These relatively time-consuming events require a long T-BsAb half-life, and a short one might lead to insufficient antitumor efficacy. To overcome this problem, some researchers add an Fc fragment or another large protein, like human serum albumin, to low molecular weight T-BsAbs, resulting in a longer half-life [40,41,42,43,44]. Moreover, preserving the neonatal FcR (FcRn)-binding activity of the Fc fragment prolongs the circulation time of T-BsAbs, while abolishing FcγR-binding ability reduces the risk of off-target events [45,46,47]. In ongoing clinical trials, T-BsAbs with a high molecular weight (>150 kDa) tend to be administered at most once a week.

Based on previous experience with conventional antibody therapeutics, it is logical that the affinity of a T-BsAb to a TAA has a great influence on its distribution [48,49,50]. Although the low affinity of antibody agents results in poor tumor accumulation, excessively high affinity leads to poor penetration within tumor tissues and rapid clearance from the tumor, resulting in insufficient distribution. As with TAAs, the binding affinity to CD3 expressed on T cells has significant effects on the biodistribution of T-BsAbs. Mandikian et al. generated several anti-HER2 T-BsAbs, each with an anti-CD3 moiety with a different binding affinity (CD3εH and CD3εL in order of strength), and evaluated their biodistribution in mouse models. Control T-BsAbs that bound CD3 with various affinities but did not bind HER2 failed to show selective accumulation in either HER2-positive or -negative tumors. Conversely, distribution to secondary lymphoid tissues (spleen and lymph nodes) was observed in proportion to the binding affinity to CD3. Therefore, the authors anticipated that a T-BsAb with relatively low affinity to CD3 might result in efficient tumor accumulation without sequestration in T cell-rich tissues. Indeed, an anti-HER2/CD3εL T-BsAb exhibited greater distribution to HER2-positive tumors than an anti-HER2/CD3εH T-BsAb. Notably, there were no differences between these two T-BsAbs in terms of accumulation in HER2-negative tumors, demonstrating that relatively high affinity to CD3 could hamper the effective tumor targeting of T-BsAbs due to CD3-mediated trapping in secondary lymphoid tissues by strong binding to T cells localized there [51].

The PK of T-BsAbs may be critically affected by their concentration as well as by the target affinity. A biodistribution study conducted by List and Neri showed that their T-BsAb could selectively accumulate in TAA-positive tumors without trapping events by peripheral T cells. These data, coupled with PK modeling analysis, indicated that this selective targeting could be achieved only when the T-BsAb was administered at a concentration below the dissociation constant K_D_ for CD3 binding [52]. Thus, it may be good practice to use T-BsAbs at a concentration lower than the anti-CD3 K_D_ value. However, since the T-BsAb used in the study had a rather low affinity to the CD3 molecule (K_D_ of 200 ± 78 nM), further studies are needed to determine whether this theory is true in settings where a T-BsAb has a relatively high affinity to CD3.

The presence or absence of abundant T cells in the tumor microenvironment might determine the T-BsAb distribution within tumor tissues. Waaijer et al. found that [^89^Zr]Zr-N-suc-Df-ERY974 (a ^89^Zr-labeled anti-glypican 3 T-BsAb) was preferentially distributed in stromal regions with high numbers of CD3^+^ T cells within HepG2 tumors in human immune cell-engrafted mice. This selective distribution was also observed in the spleen and mesenteric lymph nodes, which were both rich in human CD3-positive cells. It is well known that inflamed tumors with marked T cell infiltration are associated with a better prognosis than not only immune-desert tumors absent of T cells, but also immune-excluded tumors that cannot be infiltrated by T cells despite these cells’ presence around the tumor bed [53]. Therefore, the pharmacokinetic distribution of T-BsAbs may depend on the locations of T cells within tumors in addition to T cell accessibility. However, it is notable that the pre-existence of T cells in tumors promotes T-BsAb infiltration, and vice versa; that is, T-BsAbs can facilitate T cell migration and infiltration into tumors. The presence of T cells in tumors before T-BsAb therapy is definitely a predictive parameter for good outcomes. The relationship between T-BsAb kinetics and T cell dynamics are discussed in detail below.

### 2.2. Redirection of Antitumor Immunity

T-BsAbs are referred to by many other names, such as T cell-engaging BsAbs or T cell engagers, T cell-dependent BsAbs, T cell-redirecting BsAbs, T cell-recruiting BsAbs, etc. These terms are all derived from the ability of T-BsAbs to induce an antitumor immune response via internal T cell cytotoxicity. Indeed, many preclinical studies demonstrated that T-BsAbs obviously increased the quantity of activated tumor-infiltrating or tumor-surrounding T cells after treatment, accompanied by effective tumor growth inhibition [54,55,56,57]. This active immune-redirecting activity is not shown by conventional immuno-oncology therapies. This includes CPIs, which release immune tolerance signals and which in terms of efficacy are heavily dependent on the intrinsic immunogenic properties, meaning the hot or cold tumor, at the pretreatment status. Therefore, T-BsAbs are expected to be highly beneficial for patients who are insensitive or resistant to traditional immune therapies. Furthermore, several pharmacological studies investigated maximizing T-BsAb abilities, through means such as improved T cell trafficking and modulated T cell activation, and identified better designs that enable T-BsAbs to more effectively redirect T cells against tumors [58,59]. Questions remain, however. For instance, what populations of T cells are redirected, and how do T-BsAbs recruit peripheral T cells into tumors? Our understanding of the precise immune mechanism of T-BsAbs at both the cellular and molecular levels is still insufficient to allow us to develop more effective agents. In this section, we have introduced recent advances regarding the T cell-redirecting activity of T-BsAbs.

In the typical adaptive immune response, T cell priming is initiated by the combination of signals resulting from engagement of the TCR/CD3 complex to the MHC/peptide complex (signal 1) and from the interaction between costimulatory receptors, such as CD28 and CD137, and their ligands (signal 2). In contrast, T-BsAbs have been reported to be able to induce T cell activation by bridging to a tumor cell in a costimulation-independent manner. This capability is explainable by the relatively strong affinity of T-BsAbs to the T cell populations that they activate.

Former explanation is based on the efficiency of IS formation. As T-BsAbs typically have a stronger affinity for TAA and CD3 (K_D_ in the nanomolar range) than for TCR-MHC/peptide engagement (K_D_ in the micromolar range) [60], they easily crosslink TAAs to specific CD3s, and many TCR/CD3 complexes tend to be deposited at the interface between two cells. This clustering of the activation receptors leads to the efficient formation of IS, resulting in T cell activation via only signal 1 [61].

On the other hand, latter on is based on the subset analyses of activated T cell populations, as discussed below. In contrast with the antigen-presenting cell-mediated process, which mainly activate naïve T cell subsets, T-BsAbs seem to activate memory T cells instead of naïve T cells. In vitro analysis revealed that an anti-CD19×CD3 BsAb primarily activated CD45RO-positive T cells, which are typically memory T cells including central memory and effector memory phenotypes, and that these cells showed highly cytotoxic activity among CD8-positive peripheral T cells. On the other hand, naïve T cells with a CD8/CD45RA-double positive phenotype did not induce tumor cell lysis [62]. Consistent with these findings, another study demonstrated that both T_EM_ and T_EMRA_ subsets had stronger cytotoxicity than naïve T cells [63]. The authors conducted gene expression analysis and found that these memory T cells showed a relatively high expression of genes associated with CD8^+^ T cell function, including *PRF1*, *GZMB*, and *LILRB1*. So far, there is no defined understanding regarding the activity of effector T cells engaged by T-BsAbs presumably due to the too short-term effector status to investigate it. Considering that T-BsAb can lead systemic T cells, which is expected to include both naïve and memory T cells, to be less effective over time shown by Meermeier et al., however, effector T cells would experience more rapid exhaustion [64]. Together, these studies suggest that preferential activation of memory T cells could explain why CD3-mediated signaling (signal 1) can initiate T cell cytotoxicity in T-BsAb-anchored settings in the absence of signal 2.

The second question regarding the pharmacodynamics of T-BsAbs is how they increase the amount of tumor-infiltrating T cells after treatment. As mentioned above, T cell enrichment within tumor masses has been widely reported, and this dynamic has also been observed even when there are very few immune cells within xenografted tumors before T-BsAb treatment [54,65]. One study demonstrated T-BsAb-induced T cell recruitment by simultaneously examining in vivo T cell trafficking and visualizing T-BsAb kinetics in mouse models. The authors labeled isolated T cells and their T-BsAb with two separate dyes that fluoresced at different wavelengths in order to distinguish between antibodies and T cells. They observed co-localization of their T-BsAb and T cells in tumors, and fluorescent signals from T cells peaked at 192 h after T-BsAb administration [66]. While these reports indicated an increased T cell quantity within tumors using in vivo imaging systems, several mechanistic studies have been performed to elucidate the cellular and molecular mechanism of the events involved. Based on these studies, it is considered that T cell redirection by T-BsAbs is caused by two types of activities, specifically T cell proliferation in the tumor mass and recruitment from the periphery to tumor tissues.

T cell proliferation initiated by crosslinking with tumor cells via T-BsAbs has been demonstrated both in vitro, by evaluating the dilution rate of T cell-labeling dyes like CFSE [67,68], and in vivo, by measuring the expression of Ki67 (a proliferation marker) in intratumoral T cells after treatment [69,70]. Notably, the T cell proliferation reported in these studies was undoubtedly induced by T-BsAbs, as control BsAbs did not elicit a comparable level of proliferation. However, despite the fact that proliferation contributes to T cell enrichment in tumors, T cell recruitment seems to be the major mechanism involved. An ex vivo study revealed that tumor accumulation of CD8^+^ T cells induced by an anti-HER2 T-BsAb was suppressed to physiological levels by co-administration of a sphingosine 1-phosphate receptor agonist, which inhibits lymphocyte egress from secondary lymphoid organs and blocks trafficking between blood and tissue [71]. This indicates that T cell proliferation could not compensate for the lack of T cell recruitment [72]. In addition, the authors showed important data about the molecular mechanism of T cell recruitment by the anti-HER2 T-BsAb. They found that T-BsAb treatment induced the expression of various pro-inflammatory cytokines and chemokines in tumors, including the CXCR3 ligands CXCL9, CXCL10, and CXCL11, and upregulated CXCR3 expression on T cells. It is known that this chemokine axis is a main regulator of T cell migration and the expression of these ligands is induced by IFNγ [73,74]. Thus, the authors evaluated the impact of neutralizing antibodies for IFNγ and CXCR3 on T-BsAb efficacy and showed that both antibodies were able to inhibit T cell recruitment into tumors and attenuate the inhibition of tumor growth by T-BsAb. The authors therefore concluded that T-BsAb-induced T cell recruitment depends on the chemokine axis of CXCR3 and its ligands and requires IFNγ secretion from activated T cells.

To fully understand T-BsAb activity, it is necessary to elucidate the spatiotemporal mechanism of T cell recruitment in addition to the molecular mechanism. Groeneveldt and colleagues posed an important question about T-BsAbs: does a T-BsAb first bind a tumor cell and then bridge with a preexisting T cell, or does it first bind a T cell in the lymphoid tissues or circulation and then just bring it to the tumor tissues? This issue is important because if the latter is true, it means that T-BsAbs could recruit peripheral T cells into a tumor even if the tumor contains no preexisting T cells. Although this question remains unanswered, another critical question, namely, whether resident intratumoral T cells must be present before T-BsAb treatment in order to further recruit peripheral T cells into the tumor, has been studied in a humanized mouse model [75]. Cremasco et al. prepared two separately labeled T cell populations with the aim of distinguishing resident T cells, which were intradermally inoculated with cancer cells, from recruited T cells, which were intravenously injected as peripheral T cells. The authors then evaluated cell dynamics in tumor-bearing mice with an escalating ratio of T cells to tumor cells. After xenografting of a 1:1 proportion of T cells and tumor cells, a number of recruited T cells were detected in the tumor at 72 h post T-BsAb treatment. However, when no T cells were inoculated with resident T cells (i.e., cancer cells), the number of recruited T cells was significantly lower, and equivalent of that in the vehicle group. Of note, with lower proportions of resident T cells at baseline, such as 1:10 or 1:100, the T-BsAb was able to induce T cell recruitment from the periphery but the number of recruited cells was lower than when the ratio was 1:1. These findings suggest that a certain number of pre-existing T cells in the tumor is essential for initial inflammation and subsequent peripheral T cell recruitment. Supporting this idea, we performed simultaneous in vivo imaging of a T-BsAb and T cells as in the aforementioned system [66] and confirmed that peak T cell accumulation occurred after the T-BsAb was delivered to the tumor (data not shown). Our results revealed that the T-BsAb first contacted the tumor cells and then brought in peripheral T cells. Altogether, it is reasonable to assume that the following process occurs after T-BsAb administration: (i) the T-BsAb is first delivered to the tumor tissue, where it crosslinks a tumor cell and a preexisting T cell; (ii) crosslinking induces T cell activation and causes the tumor to develop the inflamed phenotype, exemplified by active secretion of cytokines and chemokines; and (iii) peripheral T cells that express CXCR3 are recruited toward the tumor tissue according to the concentration gradient of pro-migration factors

### 2.3. Local Mechanism of Action within Tumor Tissues

After delivery to a tumor tissue, T-BsAb crosslinks a tumor cell with a resident T cell or a subsequently recruited T cell, depending on the bispecific property of the T-BsAb. Then, an IS is formed in the interface between the two cells, and it transduces a T cell activation signal. Last, the activated T cell attacks the bridged tumor cell and causes its death. Moreover, it has been reported that T-BsAb-activated T cells can achieve serial killing of neighboring tumor cells as a result of subsequent random contacts [76]. A series of these processes are considered to comprise the typical T-BsAb mechanism (mode) of action (MOA) within tumor tissues. Recently, many conceptual studies of this MOA have been conducted and the processes involved are being clarified more precisely.

An IS is a circular, supramolecular structure that forms at the interface between an antigen-presenting cell and a T cell when a TCR is engaged with a peptide-loaded MHC molecule in physiological conditions [77,78]. Furthermore, an IS is formed between a T cell and a tumor cell when the tumor cell presents neoantigens via MHC molecules and is recognized by the T cell. Therefore, it is not surprising that T-BsAbs promote IS formation after crosslinking a tumor cell and a T cell [79]. Initial work on T-BsAb-induced ISs investigated the molecular composition of both conventional and T-BsAb-induced ISs by comparing multiple protein markers distributed in the organized structure. Researchers have found that the markers in T-BsAb-induced ISs are extremely similar to those seen in conventional ISs [22]. Further, these markers have been commonly detected in three types of ISs induced by T-BsAbs with different configurations, indicating that this might be a general pattern among T-BsAbs. Many studies have visualized T-BsAb-induced ISs in vitro [80,81,82]. Recently, IS formation was successfully evaluated by Cremasco et al. in an in vivo humanized mouse model involving transfer of human hematopoietic stem cells [75]. They applied multiphoton intravital microscopy and assessed IS formation from three points of view: T cell dynamics (speed and movement direction), time of interaction between a tumor cell and a T cell, and contact area. Using this system, the authors revealed that their T-BsAb induced rapid and lasting IS formation that resulted in tumor cell killing and T cell proliferation.

The configuration of T-BsAbs seems to have a large influence on IS formation after crosslinking of a tumor cell and T cell. As a T-BsAb-induced IS is compositionally similar to that formed by the association between a TCR and MHC/peptide complex [22], the distance between two antigens targeted by T-BsAbs was estimated to be similar to the length of the TCR-MHC/peptide complex [83,84]. This hypothesis was proved by a study that evaluated the relationship between the epitope distance to the cell membrane and the potency of target cell lysis by T-BsAbs [85]. This result is consistent with that of a subsequent study of the relationship between epitope proximity and the efficiency of IS formation [86]. In order to achieve an optimized T-BsAb, Wuellner et al. added two TAA-binding domains to either the N-terminus or C-terminus of an anti-CD3 IgG antibody, and compared their cytotoxicity. Their in vitro analysis indicated that the N-terminally fused antibody caused more potent cell lysis [87]. In contrast, Santich et al. evaluated the optimal location of the CD3-binding domain within the anti-TAA IgG antibody. They prepared three dual bivalent T-BsAbs with CD3-binding domains, a fused light chain C-terminus, a fused heavy chain C-terminus, or a replaced CH1 domain, and compared their antitumor efficacy. The T-BsAb with the light chain-fused anti-CD3 domain showed the best results, and the authors concluded that this spatial configuration is the most suitable to induce robust antitumor responses presumably because the interdomain distance as long as a single Ig fragment (CL) is the best length for bridging of a TAA and a CD3 [88].

Studies of the molecular mechanism of T-BsAb-mediated cell killing have identified factors that do or do not contribute to cytotoxicity after IS formation. One in vitro study showed that T cells activated by a T-BsAb utilized perforin and granzyme A/B to cause necrosis and apoptosis, respectively, when bridging tumor cells [89]. While perforin-induced necrosis was observed regardless of the status of the tumor cell cycle, granzyme-mediated apoptosis was likely to be dependent on the proliferative status, indicating greater sensitivity of proliferating cells to granzyme A/B. Furthermore, the authors demonstrated that the Fas-Fas ligand (FasL) pathway did not contribute to the T-BsAb-mediated cytocidal effect against Hodgkin’s derived cell lines despite the upregulation of FasL expression in activated T cells. In an inhibition assay, Gruen and colleagues found that other death ligands in addition to the Fas-FasL pathway, including TRAIL and TNFα, also failed to cause T-BsAb-induced lysis of B-cell lines from leukemia and lymphoma [90]. Recently, however, we demonstrated that secreted cytokines from T-BsAb-activated T cells damaged target cells in a cell contact-independent manner, although cell contact-dependent tumor cell killing, which was presumably attributed to perforin and granzyme activity, showed stronger cytotoxicity [82]. This discrepancy might be explained by target cells having differing sensitivity to various ligands and cytokines. Future research is expected to elucidate the mechanism underlying differences in sensitivity and to identify appropriate tumors for T-BsAb treatment.

As cell contact-independent cytotoxicity caused by death ligands or cytotoxic cytokines results in tumor cell death independently of the T-BsAbs target antigen, T-BsAbs are expected to induce off-target cell killing against neighboring cells that do not express targeting TAAs in solid tumors. This additional cytotoxic activity, called the bystander effect, is well known in the context of treatment with radiation and antibody-drug conjugates (ADCs). The potency of the bystander effect might significantly increase clinical efficacy, particularly in the context of ADCs, as exemplified by the finding that trastuzumab deruxtecan (T-DX), an ADC that was proved to cause the bystander effect in preclinical settings, showed favorable results in a clinical trial of patients with gastric cancer even though trastuzumab emtansine (T-DM), which targets the same TAA as T-DX (HER2) but does not induce the bystander effect, showed no clinical effectiveness [91,92]. Similarly to ADC, bystander effect is also considered beneficial for T-BsAb therapy especially against the tumors with heterogeneous TAA expression, though its relevance to toxicity remains to be explored yet. Fortunately, as confirmed by Ross et al. in preclinical experiments [93], T-BsAbs also seem to cause the bystander effect. They found that EGFR-negative tumor cells that were not sensitive to EGFR/CD3 BiTE were efficiently lysed when co-cultured with EGFR-positive tumor cells. Correspondingly, this off-target cell killing was also demonstrated in in vivo experiments that showed marked elimination of mixed xenograft tumors consisting of EGFR-positive and -negative cells. The authors concluded that this bystander effect was caused by upregulation of ICAM-1 and FAS on the tumor cells in response to proinflammatory cytokines, resulting in IS- and FASL-mediated cell lysis, respectively. These favorable data will encourage the T-BsAb treatment of refractory tumors with heterogeneous expression of the target TAA

## 3. T-BsAbs in Development for Solid Tumors

Recently, increasing numbers of T-BsAbs have entered development for the clinical treatment of not only hematologic cancers but also solid cancers. However, in contrast to CPIs, only a small percentage of T-BsAbs in current clinical trials target solid tumors [94]. Moreover, significantly fewer T-BsAbs have been developed compared to CPIs and CAR-Ts. Given that relatively more T-BsAbs have been clinically studied for hematologic tumors, in which tumor cells and T cells colocalize, future development should increasingly focus on understanding T-BsAb pharmacology in solid tumors.

### 3.1. Preclinical Research

Before T-BsAbs are investigated clinically, many strategies to increase efficacy and safety are undertaken in preclinical research. One focal point has been to optimize the T-BsAb format that most effectively induces potent tumor killing while minimizing various side effects. Indeed, there is considerable variation in the format of BsAbs when not limited to T cell-engaging BsAbs [95,96]. Consistent with this, diverse types of T-BsAbs that target various TAAs expressed on tumor cells have been developed in preclinical settings so far. Here, we introduce some preclinical data of promising T-BsAbs while highlighting their unique technologies or advantages (Table 1).

Human epidermal growth factor receptor 2 (HER2), which is frequently overexpressed in a wide range of human cancers, is one of the most commonly targeted antigens, not only by monoclonal antibodies, but also by T-BsAbs. In 2014, Junttila and colleagues reported a novel anti-HER2 T-BsAb (HER2-TDB: T cell-dependent bispecific antibody) with an IgG format. HER2-TDB induced polyclonal T cell activation and proliferation only when HER2^+^ tumor cells were co-incubated with T cells, and consequently caused tumor cell death. The authors also found that HER2-TDB activity correlated with target cell HER2 expression levels, and anticipated that only 1000 HER2 molecules, or 1% occupancy on the cell surface, are required to induce T cell-mediated killing. In vivo tumor growth inhibition was demonstrated in both immunodeficient mice and huHER2-transgenic mouse models [97].

Two additional anti-HER2 T-BsAbs are characterized by their unique formats. The first T-BsAb was generated by dimerizing a monovalent heavy/light chain pair against HER2 and a single chain unit against CD3 in an asymmetrical conformation [98]. This unique format provides an advantage in the purification of the targeted heterodimer because it has a different molecular weight than homodimers. The second T-BsAb consists of anti-HER2 IgG and two anti-CD3 scFvs fused to the C-terminus of IgG light chains [99,100]. The nature of this bivalent targeting ability has often been discussed. It was reported that multivalency for TAAs strengthens the binding avidity of T-BsAbs to a tumor cell, resulting in enhanced cytotoxic potency and specificity [101,102,103]. On the other hand, the necessity of a multivalent CD3-binding arm is still controversial. As monovalent engagement with CD3 is sufficient to induce T cell activation, there is a concern about off-target T cell activation via crosslinking of CD3 molecules. As with avidity to TAAs, however, it is expected that multivalency for CD3 promotes active CD3 engagement. Moreover, some previously reported T-BsAbs, as well as those in our research, did not induce T cell activation in the absence of TAA engagement despite the presence of bivalent anti-CD3 arms [104,105,106,107]. Although it is yet unclear whether these T-BsAbs have simultaneous bivalency for CD3, that is a capability to bridge two CD3 molecules, bivalent binding of CD3 alone seems to be unable to set up the condition where T cells are activated in a tumor cell-crosslinked manner. Therefore, the most important discussion seems to be whether two CD3-binding arms can bind to two CD3 molecules simultaneously, thereby crosslinking them. Generally, lack of simultaneous binding to CD3 is preferred due to the aforementioned reason.

Although HER2 is frequently overexpressed in breast cancer and gastric cancer and is commonly used as a target antigen, its drawback is that it is expressed on normal tissues as well [108,109]. On-target, off-tumor engagement of anti-HER2 T-BsAbs may cause severe side effects, which dissuades us from utilizing HER2 for T cell-engaging immunotherapy. To address this issue, Ruiz et al. developed a T cell bispecific antibody (TCB) that targets p95HER2, a carboxyl-terminal fragment of HER2, instead of full-length HER2 [110]. According to the authors, approximately 40% of HER2^+^ tumors expressed p95HER2, while its expression was not detected in normal tissue samples. Their data showed that p95HER2-TCB induced T cell activation and target cell death against p95HER2^+^ tumor cells but not against HER2^+^/p95HER2^-^ tumor cells or nontransformed cells. This novel strategy for targeting a tumor cell-specific antigen seems to have great potential for developing T-BsAbs with fewer treatment-related adverse events.

In addition to HER2, many T-BsAbs target epithelial growth factor receptor (EGFR) and its active mutant EGFRvIII, both of which are frequently detected in glioblastoma [111,112]. ATTACK (Asymmetric Tandem Trimerbody for T cell Activation and Cancer Killing) is a single-chain anti-EGFR T-BsAb with trivalent EGFR binding and monovalent CD3 binding [113]. With its intermediate molecular weight of ~100 kDa, ATTACK may have two advantageous properties that promote a long half-life in the circulation and the efficient penetration of tumor tissues, which are issues seen with small T-BsAbs of around 50 kDa and large T-BsAbs of more than 150 kDa, respectively. ATTACK also features oppositely oriented antigen-targeting moieties. This structure facilitates the simultaneous binding to EGFR on tumor cells and to CD3 on T cells, and effectively induces IS formation. As a result, early signaling downstream of the TCR promotes T cell activation and leads to lysis of EGFR-positive tumor cells.

To achieve similarly efficient crosslinking between tumor cells and T cells, we used another approach involving rearranging the domain order of variable fragments. Although studies have examined the effect of the configuration of antigen-binding domains on T-BsAb effectiveness, and it is known that the length between two paratopes for a TAA and CD3 is critical for IS formation (discussed below), the importance of the domain order of variable fragments has rarely been considered. We found that there was a suitable domain order for crosslinking of two antigens and T cell-mediated cytotoxicity in both IgG-like and small diabody formats [106,114]. 

In addition to EGFR and EGFRvIII, epithelial cell adhesion molecule (EpCAM) and carcinoembryonic antigen (CEA) are common antigens that have been targeted by T-BsAbs [115,116,117,118,119,120]. However, the demand for additional tumor-specific antigens is increasing in order to mitigate on-target toxicity to normal tissues. Here we concisely introduce some examples of T-BsAbs that are in preclinical development and that target highly tumor-specific antigens. T-BsAbs targeting a complex of a glycan and a protein, such as proteoglycans and glycoproteins, have been drawing increasing attention. Glypicans are a family of six heparan sulfate proteoglycans in vertebrates, and some have found to be expressed specifically in cancer [121]. The tumor-specific glypicans that have been utilized in T-BsAbs are glypican 1 and glypican 3 [55,122]. Mucin 16 is an example of a membrane glycoprotein targeted by a T-BsAb [123]. Additionally, some groups have taken advantage of members of the B7 family that are overexpressed in human cancers and are related to tumor immunity. T-BsAbs that target B7-H3, B7-H4, or B7-H6 have shown promising antitumor effects in mouse models against melanoma, breast cancer, and ovarian cancer, respectively [124,125,126]. Other distinctive target antigens include a lyase, a Wnt signaling regulator, an orphan receptor, and a sialylated cluster of differentiation (CD) antigen [57,127,128,129]. Several T-BsAbs mentioned here are now in clinical trials for evaluation of their efficacy and safety.

Another preclinical strategy is to take advantage of combination therapy utilizing T-BsAbs and various agents such as CPIs, costimulatory agonists of T cells, and oncolytic viruses. So far, increased expression levels of immune checkpoint molecules on both tumor cells and T cells have been reported as a major mechanism of resistance to blinatumomab therapy [130,131,132]. Consistent with this finding, many preclinical studies reported that T-BsAb therapy in combination with CPI therapy has synergistic effects against hematological as well as solid tumor models, although some of them were already investigated clinically. Some groups reported that administration of anti-PD-L1 antibody reinforced the antitumor efficacy of several T-BsAbs, including anti-HER2/CD3, anti-CEA/CD3, and anti-CD20/CD3 [70,97]. Another group reported that the combination of anti-PD-1 antibody and anti-GUCY2C/CD3 T-BsAb obviously delayed tumor regrowth [57]. These data indicate that blockade of the PD-L1/PD-1 pathway plays a key role in improving the efficacy of T-BsAbs. However, some studies have failed to show evidence of this synergistic activity, even when using T-BsAbs targeting HER2 molecules [99,133]. One authors reasoned their T-BsAb itself could overcome PD-1/PD-L1 checkpoints by its high avidity, while another authors insisted on the ineffectiveness of PD-1/PD-L1 axis-specific blockade in their settings. Recently, Guo and colleagues demonstrated that blockade of another immune checkpoint molecule, TIM-3, enhanced the antitumor activity of an anti-EpCAM/CD3 T-BsAb derived from γδ T cells [134]. This finding shows that resistance mechanisms other than the PD-L1/PD-1 pathway may exist, and may vary depending on tumor types and T-BsAbs. Further translational research is necessary to clarify resistance that limits the use of T-BsAbs against solid tumors.

Claus et al. developed a 4-1BB ligand-fused TAA-targeting Fab (TA-4-1BBL) to introduce costimulatory signals dependent on the presence of tumor cells into T cells. They adopted fibroblast activation protein (FAP), which is expressed in the tumor stroma as a TAA, and evaluated the combination therapy of FAP-4-1BBL and anti-CEA/CD3 T-BsAb. In vitro analysis showed that this combination therapy caused potent T cell activation and cytokine secretion in comparison with T-BsAb monotherapy. In CEA-positive tumor cells and a FAP-positive fibroblast mixture tumor model, combination therapy markedly suppressed tumor growth whereas T-BsAb monotherapy failed. In addition, immunohistochemistry analysis demonstrated greater T cell accumulation in the tumor mass in the presence of 4-1BB activation signaling [135]. Skokos and colleagues combined T-BsAb therapy with CD28 signaling instead of 4-1BB signaling. They prepared anti-PSMA/CD28 and anti-MUC16/CD28 BsAbs as partners of anti-PSMA/CD3 and anti-MUC16/CD3 T-BsAbs, respectively. Unlike in the previous research with TA-4-1BBL, they intended to introduce both TCR/CD3-mediated and CD28-mediated activation signals to T cells through the same TAAs. Despite this difference, the results of the combination therapy corresponded with those derived using TA-4-1BBL, with greater cytokine secretion and more efficient antitumor activity compared to monotherapy [80]. These data indicate that T-BsAbs may have increased therapeutic efficacy when combined with costimulatory agonists.

The last notable modality that has been combined with T-BsAbs is oncolytic viruses. One approach utilizes tumor-specific viruses as a delivery tool for T-BsAbs. By inserting genes coding for full-length T-BsAbs into the virus genome, oncolytic viruses can express and secrete each T-BsAb only within tumor tissues. Of note, T-BsAb-armed oncolytic viruses induced accumulation and retention of tumor-infiltrating T cells, and their antitumor efficacy was superior to those of the parent viruses whereas any significant toxicity was observed in mice [136,137]. Despite the lack of safety data in human regarding T-BsAb-armed oncolytic viruses, the favorable safety profile of simple oncolytic viruses has been warranted in cancer patients [138,139]. Furthermore, the authors expected localized T-BsAb expression by onctolytic viruses would reduce the adverse events like cytokine release syndrome (CRS) known to occur during T-BsAb circulation. Another approach intends to enhance T-BsAb efficacy by converting tumors with an immune desert, referred to as cold tumors, into inflamed tumors, namely, hot tumors. Although it is considered that T-BsAbs themselves have the ability to promote T cell infiltration into tumors (described in detail later), some tumors show insensitivity to T-BsAb therapy due to their immune deficiency. To overcome this problem, Groeneveldt et al. utilized an oncolytic reovirus to cause an immune reaction specifically within the tumor tissues. They demonstrated that intratumoral administration of replication-competent reovirus induced an interferon response, including the expression of T cell-attracting chemokines CXCL10 and CCL5, which promoted T cell recruitment into the tumors. Furthermore, pretreatment with reovirus significantly enhanced the antitumor efficacy of subsequent T-BsAb therapy against the subcutaneous KPC (*Kras^G12D/+^*; *Trp53^R172H/+^*; *Pdx1-Cre*) tumor model and the orthotopic HER2-positive breast cancer model. Importantly, reovirus treatment increased sensitivity to the T-BsAb treatment not only in local tumors (virus-injected), but also in distant tumors (non-injected). The authors concluded that intratumoral administration of reovirus has a systemic effect that converts cold tumors into hot tumors, and this pretreatment might be also be effective in conjunction with T-BsAb therapy against metastatic cancers [140].

**Table 1:** The current T-BsAb strategies in preclinical development.

Strategy	Executive Summary	Reference
Antigen Selection	• HER2, EGFR, EpCAM, and CEA are common antigens that have been targeted by T-BsAbs.	[97,111,116,120]
	• Aimed at mitigating on-target toxicity to normal tissues, more tumor-specific antigens, such as mutant proteins and complexes of a glycan and a protein, have been exploited as a T-BsAb target.	[110,122,123]
Format Selection	• Asymmetrical conformation consisting of an anti-TAA moiety and an anti-CD3 moiety provides an advantage in the purification of the targeted heterodimer.	[98]
	• Although multivalency of T-BsAbs for a TAA is beneficial for its potency and specificity, one for a CD3 has pros and cons.	[102,104]
	• A suitable domain order of variant fragments brings about efficient crosslinking of two antigens and T cell-mediated cytotoxicity.	[106,114]
Combination therapy	• T-BsAb therapy in combination with CPI therapy has synergistic effects against multiple types of tumor, including hematological and solid tumors.	[57,70,97]
	• Inducing costimulatory signals with an agonist, CD28 or 4-1BB ligand, strengthens therapeutic efficacy of T-BsAbs.	[80,136]
Utilization of oncolytic virusis	• Tumor-specific viruses can be applied as a delivery tool for T-BsAbs	[137,138]
	• Pretreatment with oncolytic virus promotes T cell infiltration by inducing immune reactions within tumor tissues, which results in enhanced antitumor activity of subsequent T-BsAb therapy.	[141]

### 3.2. Clinical Research

In the last 5 years, more than 30 different T-BsAbs have been studied as monotherapy for solid tumors in clinical trials (Table 2). Catumaxomab, which binds EpCAM on tumor cells and CD3 on T cells while retaining affinity to FcγR-positive cells, was the first clinically successful T-BsAb and was approved as a therapeutic agent for malignant ascites by the European Union in 2009 [141,142,143]. Since then, catumaxomab has been evaluated in terms of efficacy and safety against various solid tumors, such as non-small cell lung cancer, ovarian cancer, and gastric cancer [144,145,146,147,148]. In phase II studies, catumaxomab showed disappointing results in patients with platinum-resistant epithelial ovarian cancer [145]. Similarly, in the postoperative setting prior to standard chemotherapy for epithelial ovarian cancer, no remarkable results were observed (complication rate of 51%) [147]. On the other hand, researchers found that catumaxomab as adjuvant therapy could induce T cell activation and migration to peripheral tissues against gastric cancer, consequently leading to secondary antitumor immune responses to various tumor antigens other than EpCAM [146]. Moreover, catumaxomab demonstrated relatively favorable efficacy against gastric cancer in the postoperative setting (complication rate of 33%). However, development of this agent was terminated mainly due to severe toxicity caused by Fc-mediated, off-target T cell activation and the high immunogenicity of the non-human IgG backbone [149], and catumaxomab was finally withdrawn from the market for commercial reasons in 2017. However, catumaxomab has since been studied for patients with advanced gastric carcinoma with peritoneal metastasis in China, and it is expected to become available again. Another early T-BsAb in the field of solid tumor treatment was ertumaxomab, which is a mouse-rat hybrid BsAb that like catumaxomab, targets HER2 and CD3. Although this agent showed encouraging clinical responses in some patients with metastatic breast cancer [150], it was not approved, also due to unacceptable toxicity.

Currently the most advanced T-BsAb is a CEA T cell bispecific antibody (CEA-TCB) named cibisatamab. This molecule has a bivalent CEA-binding Fab domain and a monovalent CD3-binding Fab domain within the IgG format. This asymmetric structure gives it stronger binding avidity to CEA than to CD3, providing high tumor cell specificity. Cibisatamab was studied for locally advanced and/or metastatic CEA-positive solid tumors in phase Ia/Ib studies, and preliminary results reported that monotherapy resulted in evident antitumor activity with manageable adverse events [151]. Moreover, its efficacy seemed to be enhanced by combination with the anti-PD-L1 CPI atezolizumab. Cibisatamab is now being evaluated in combination with atezolizumab in patients with colorectal cancer or non-small cell lung cancer. 

Prostate-specific membrane antigen (PSMA) is overexpressed in almost all stages of prostate cancer, and is the most common TAA targeted by T-BsAbs under development. Six types of PSMA-targeting T-BsAbs have been developed for prostate cancer in the past 5 years, and one has been developed for advanced-stage solid tumors. Among them, pasotuxizumab (PSMA-targeting BiTE: Bispecific T cell Engager) is the only T-BsAb for which the phase I clinical study is already complete. In the study, researchers assessed the safety and maximum tolerated dose (MTD) of pasotuxizumab in patients with advanced, castration-resistant prostate cancer. The treatment was well tolerated, other than the emergence of anti-drug antibodies observed in the subcutaneous injection cohort. In addition, pasotuxizumab was associated with a decline in PSA levels, indicating the potential efficacy of BiTE monotherapy against prostate cancer [37].

T-BsAbs are also being developed for other indications. For gastrointestinal cancers, including gastric and colorectal cancer, two phase 1 studies using MEDI-565/MGD007 have already been completed, and results of the former have been reported. According to the authors, while severe adverse events were prevented by pretreatment with dexamethasone, MEDI-565 failed to provide an objective response. Gynecologic cancer-targeting T-BsAbs have been also developed. They are designed to engage HER2 and MUC16 for the treatment of female patients with breast cancer and ovarian cancer, respectively. Other specific indications for T-BsAbs under development include glioblastoma, neuroblastoma, and small cell lung cancer.

## 4. Future Perspectives, Challenges, and Conclusions

Cancer immunotherapy is emerging as the fourth pillar of anticancer treatment and has been recognized to be essential for pharmacotherapeutics because of its innovative action and remarkable efficacy. CPIs, the first successful type of immunotherapy, are being used as first-line treatments against various types of cancer worldwide [152,153]. This revolution has spurred researchers to identify additional immunotherapeutic modalities in order to successfully treat larger numbers of people. CAR-T therapy is a cell-based immunotherapy derived by applying genetic engineering to the human T cell, and has shown dramatic efficacy against hematological malignancies, as exemplified by a complete response rate of 40% in patients with relapsed or refractory diffuse large B cell lymphoma [154]. However, CAR-T treatment of solid cancers has not yet achieved favorable results, largely due to serious adverse events, including CRS, and poor accessibility to tumor tissues. In addition, order-made cell therapy is expensive in terms of preparation and maintenance, and thus it is necessary to use off-the-shelf drugs and achieve patient-friendly costs. As a solution to these problems, T-BsAbs are expected to be easy-to-use drugs with effective immune-redirecting ability comparable to that of CAR-T therapy. In fact, it is anticipated that T-BsAbs will be delivered within tumor masses, as with other antibody therapeutics, and that they will have mild toxicity, similar to that of CAR-T therapy, though unfortunately also with less effectiveness. Therefore, in order to promote T-BsAb development as a cutting edge therapy in the future, there are some challenges that must be addressed.

Although it is anticipated that T-BsAbs might have milder toxicity than CAR-T therapy, T-BsAbs might also cause severe CRS when used for treatment of not only hematological cancers but also solid cancers [37,155,156]. Therefore, some studies have sought to develop safer T-BsAbs while retaining efficient cytotoxicity by selecting T-BsAbs with the highest affinity to CD3 or with the optimal clone of the anti-CD3 arm [59,157]. These studies successfully generated improved T-BsAbs with minimal cytokine release and robust TAA-dependent cell killing and antitumor effects. The concept of persistent efficacy with less cytokine release was endorsed by another report, suggesting that cytokine release is unnecessary for T-BsAb-induced cytotoxicity [158]. However, while secreted cytokines such as TNFα might be unnecessary for direct efficacy, some cytokines contribute to additional actions, including cell contact-independent cell killing and the bystander effect. Thus, T-BsAb-induced cytokine release may be a double-edged sword and should be carefully evaluated. A previous study suggested that IL-6 produced by monocytes/macrophages played a central role in the onset and progression of CRS [159]. From this viewpoint, molecule- or cell type-specific prophylaxis and treatment, such as IL-6-directed therapy, may be quite beneficial for managing T-BsAb-induced CRS. One notable study demonstrated that an anti-IL-6R antibody ameliorated clinical CRS in a patient treated with blinatumomab [160].

Regarding tumor cell resistance to T-BsAbs, several potential causes have been discussed and reported so far. One important study regarding the resistance to blinatumomab in acute lymphoblastic leukemia showed that loss of TAA expression after treatment was due to CD81-mediated disruption of CD19 trafficking to the cell surface membrane [161]. In addition, CD19 mutation, low CD19 RNA expression, and CD19-mutant allele-specific expression can cause antigen loss on the tumor cell surface, resulting in treatment resistance [162]. As loss of expression may also occur with solid tumor-specific TAAs such as HER2 [163], resistance caused by the same mechanism as that observed with blinatumomab may also occur in solid cancers. A preclinical study in patient-derived colorectal cancer organoids found that tumors with heterogeneous and plastic expression of TAA were not eliminated as effectively as tumors with homogeneous TAA expression [164]. Beyond TAAs, CRISPR screening revealed that deficient IFNγ signaling contributed to treatment resistance [165], and that other tumor-extrinsic factors, such as the patient’s immune condition and the intratumoral vasculature, might confer critical resistance to T-BsAb therapy. In any case, we still have insufficient knowledge and experience to evaluate these potential risks, and further studies are necessary.

Data thus far suggest that T-BsAbs will be able to overcome the aforementioned challenges due to their unique properties, including CD3 affinity screening and the bystander effect. Importantly, T-BsAbs can actively redirect internal T cells and promote their recruitment from the periphery to tumor tissues just like other systems for delivering antitumor immunity. This advantage should make T-BsAb efficacy independent of the number of preexisting T cells in tumors and lead to superior results compared with immune brake-releasing CPIs. So far, a number of mechanistic findings have been reported in preclinical studies, and we have been able to regulate T-BsAb action at the molecular or cellular level. Many clinical trials are currently being conducted to evaluate the efficacy and safety of T-BsAbs, and positive outcomes are strongly expected.

In conclusion, T-BsAbs are an emerging and promising form of antibody therapeutics, and are currently under evaluation in multiple clinical trials.

## Figures and Tables

**Table 2:** T-BsAbs under clinical development for solid cancer treatment. More than 30 T-BsAbs have been investigated as monotherapies in clinical trials worldwide, most in the early phase. A variety of formats and target TAAs are being explored. Data are derived from ClinicalTrials.gov (accessed on 13 November 2021) and identifiers are shown in the table.

Cancer Type	Name	Target TAA	Format	Phase	Identifier
Solid tumor	HPN536	mesothelin	TriTAC ^1^	1/2	NCT03872206
	ERY974	GPC3	IgG-like BsAb	1	NCT02748837
	JNJ-63898081	PSMA	DuoBody (IgG-like BsAb)	1	NCT03926013
	PF-06671008	CDH3 (P-cadherin)	DART-Fc ^2^	1	NCT02659631
	MGD009/orlotamab	B7-H3	DART-Fc	1	NCT02628535
	M701	EpCAM	Fab/scFv-Fc BsAb	1	NCT04501744
	M802	HER2	Fab/scFv-Fc BsAb	1	NCT04501770
	BTRC4017A/RG6194	HER2	IgG-like BsAb	1	NCT03448042
	GEM3PSCA	PSCA	ATAC ^3^	1	NCT03927573
	AMV564	CD33	bivalent BiTE	1	NCT04128423
	GEN1044	5T4	DuoBody (IgG-like BsAb)	1/2	NCT04424641
Glioblastoma	AMG596	EGFRvIII	BiTE	1	NCT03296696
Neuroblastoma	Hu3F8-BsAb	GD2	Bivalent Fab/scFv-Fc BsAb	1/2	NCT03860207
Small cell lung cancer	AMG757	DLL3	BiTE-Fc	1	NCT03319940
NSCLC	RO6958688/RG7802/ cibisatamab	CEA	2 + 1 Fab-Fc BsAb	1/2	NCT03866239
Breast cancer	GBR1302/ISB1302	HER2	Fab/scFv-Fc BsAb	1/2	NCT03983395
NET and GIST	XmAb18087/tidutamab	SSTR2	XmAb (Fab/scFv-Fc BsAb)	1	NCT03411915
Gastric cancer	AMG199	MUC17	BiTE-Fc	1	NCT04117958
	AMG910	CLDN18.2	BiTE-Fc	1	NCT04260191
	catumaxomab	EpCAM	IgG-like BsAb	3	NCT04222114
Gastrointestinal cancer	MEDI-565/AMG211/ MT-111	CEA	BiTE	1	NCT01284231
	PF-07062119	GUCY2C	DART-Fc	1	NCT04171141
Colorectal cancer	MGD007	gpA33	DART-Fc	1	NCT02248805
Prostate cancer	AMG212/MT-112/ BAY2010112/ pasotuxizumab	PSMA	BiTE	1	NCT01723475
	HPN424	PSMA	TriTAC	1	NCT03577028
	AMG160	PSMA	BiTE-Fc	1	NCT03792841
	AMG509	STEAP1	Fab/scFv-Fc BsAb	1	NCT04221542
	ES414/APVO414/MOR209	PSMA	ADAPTIR^TM^	1	NCT02262910
	CCW702/ABBV-154	PSMA	bispecific antibody-small molecule conjugates	1	NCT04077021
	CC-1	PSMA	Bivalent Fab/scFv-Fc BsAb	1	NCT04104607
ovarian cancer	REGN4018	MUC16	IgG-like BsAb	1/2	NCT03564340

^1^ Tri-specific T cell-Activating Construct, ^2^ Dual-Affinity Re-Targeting, ^3^ Affinity-Tailored Adaptor for T Cells.

## Data Availability

Data sharing not applicable.
